# Enhanced Bounding Box Estimation with Distribution Calibration for Visual Tracking

**DOI:** 10.3390/s21238100

**Published:** 2021-12-03

**Authors:** Bin Yu, Ming Tang, Guibo Zhu, Jinqiao Wang, Hanqing Lu

**Affiliations:** 1 School of Artificial Intelligence, University of Chinese Academy of Sciences, Beijing 100049, China; gbzhu@nlpr.ia.ac.cn (G.Z.); jqwang@nlpr.ia.ac.cn (J.W.); luhq@nlpr.ia.ac.cn (H.L.); 2 National Laboratory of Pattern Recognition, Institute of Automation, Chinese Academy of Sciences, 95 Zhongguancun East Road, Beijing 100190, China; tangm@nlpr.ia.ac.cn; 3 ObjectEye Inc., Beijing 100078, China

**Keywords:** visual tracking, bounding box estimation, overlap maximization, distribution calibration

## Abstract

Bounding box estimation by overlap maximization has improved the state of the art of visual tracking significantly, yet the improvement in robustness and accuracy is restricted by the limited reference information, i.e., the initial target. In this paper, we present DCOM, a novel bounding box estimation method for visual tracking, based on distribution calibration and overlap maximization. We assume every dimension in the modulation vector follows a Gaussian distribution, so that the mean and the variance can borrow from those of similar targets in large-scale training datasets. As such, sufficient and reliable reference information can be obtained from the calibrated distribution, leading to a more robust and accurate target estimation. Additionally, an updating strategy for the modulation vector is proposed to adapt the variation of the target object. Our method can be built on top of off-the-shelf networks without finetuning and extra parameters. It yields state-of-the-art performance on three popular benchmarks, including GOT-10k, LaSOT, and NfS while running at around 40 FPS, confirming its effectiveness and efficiency.

## 1. Introduction

Generic visual tracking is a long-standing topic in the field of computer vision and has attracted increasing attention in recent decades. Despite significant progress in recent years [1,2,3,4,5,6,7,8,9,10,11], visual tracking remains challenging due to numerous factors such as very limited online training samples, large appearance variation, and heavy background clutters. In general, the single-object tracking task can be divided into two sub-tasks, i.e., localization and bounding box estimation, which aim at localizing the target roughly and predicting the precise bounding box, respectively.

In order to build an accurate tracker, the bounding box estimation branch is of great importance, since it is responsible for generating the final bounding box directly. The previous works on bounding box estimation can be roughly grouped into three categories: (1) multi-scale searching methods, (2) direct bounding box regression, and (3) bounding box estimation by overlap maximization. For the first category, conventional methods [12,13,14] could only obtain the scale of the bounding box based on the localization models, which have difficulty in estimating accurate bounding box when length–width ratio changes. For the second category, Siamese network-based methods [2,15,16] are able to predict the center, width, and length of the bounding box directly by regression. However, these Siamese tracking approaches often struggle at target localization due to the lack of online learning [17]. For the third category, bounding box estimation by overlap maximization [17] is able to be used to improve the performance of discriminative model-based trackers [18,19,20] and have shown state-of-the-art results on multiple public benchmarks [21,22,23]. Yet, the key reference information in [17], also called modulation vector, which is used to provide prior knowledge of the target, merely depends on the initial frame. This limits the robustness and accuracy of the trackers, since such reference information is biased to the initial state of the target and becomes less reliable as the frame grows, especially when the target undergoes significant variation.

**Figure 1:**
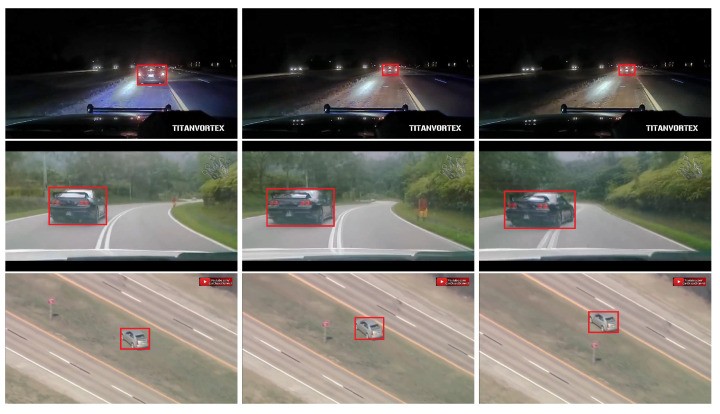
Visualization examples of targets of similar classes and close sizes, which are selected from *car-1*, *car-6*, and *car-20* on the training set of LaSOT.

To this end, we propose a novel bounding box estimation method for visual tracking, termed as DCOM, which is based on distribution calibration and overlap maximization. Inspired by [24], by taking advantage of large-scale labeled training data, we generate extra reference information by calibrating the biased distribution of the initial reference information. Specifically, we assume that every dimension in the modulation vectors follows a Gaussian distribution and observe from Table 1 that targets of similar classes and close sizes usually share similar mean and variance of the feature representations in reference information (the visualization examples of targets of similar classes and close sizes are given in Figure 1). Therefore, the mean and variance of the Gaussian distribution can be transferred across similar targets with close sizes. Then, we estimate the statistics from adequate training datasets [21,22] in advance and reuse the statistics to better estimate the distribution of reference information. More reliable and sufficient reference information can be generated from the calibrated distribution, avoiding the bias and potentially achieving more diversity of reference information. Additionally, we propose a simple yet effective updating strategy of the modulation vector to adapt the variation of the target object in online tracking. Our method is able to be built on top of off-the-shelf networks without fine-tuning and extra parameters.

In summary, our contributions are three-fold.

1. We propose a novel bounding box estimation method for visual tracking, called DCOM, which is based on distribution calibration and overlap maximization. We are the first to exploit large-scale tracking datasets on the online tracking stage by distribution calibration, creating an effective way to obtain sufficient and reliable reference information.
2. We propose a simple yet effective updating strategy of the modulation vector to improve robustness in bounding box estimation in online tracking, which cannot be implemented in previous methods.
3. Experimental results on three popular benchmarks including GOT-10k [21], LaSOT [22], and NfS [23] show that DCOM is able to improve existing state-of-the-art of trackers without bells and whistles.

## 2. Related Work

In this section, we introduce the previous bounding box estimation methods for visual tracking, which can generally be divided into three groups, i.e., multi-scale searching methods (MSS), direct bounding box regression (BBR), and bounding box estimation by overlap maximization (OM). Table 2 lists the above methods used in modern trackers.

### 2.1. Multi-Scale Searching Methods

Multi-scale searching methods are mainly utilized in traditional trackers. SAMF first introduced the multi-scale search strategy, where the final scale of the target object is selected from a scaling pool according to the response maps. DSST proposed to learn individual discriminative correlation filters for multi-scale searching efficiently. SiamFC and ECO employed SAMF and DSST to estimate the bounding box, respectively. Such methods are conceptionally simple yet computationally expensive due to the construction of image pyramids. Moreover, the prediction is coarse, since the length–width ratio is fixed.

### 2.2. Direct Bounding Box Regression

In the last decades, deep learning has developed significantly and been used in many tasks, such as detection [33], recognition [34], and localization [20]. Direct bounding box regression is also a deep-learning-based method. MDNet [26] and SiamRPN [15] are two representative trackers that both regress the bounding box directly. MDNet trained a bounding box regressor in the first frame following the settings in object detection [35]. SiamRPN utilized the RPN-based mechanism to obtain a precise bounding box. SiamRPN++ and SiamRCNN both employed an RPN-based method in SiamRPN, while SiamCAR employed an anchor-free bounding box regression. MDNet and the Siamese-based trackers only rely on the initial frame for bounding box regression, and online updating is not helpful for them considering the risk of error accumulation. Conversely, our approach enables an effective way to generate sufficient reference information and update online by distribution calibration.

### 2.3. Bounding Box Estimation by Overlap Maximization

ATOM [17] proposed an IoU-based approach, which learns to predict overlap between candidate boxes and groundtruth. In online tracking, more precise bounding box can be estimated by maximizing the overlap w.r.t. candidate boxes via gradient-ascent. DiMP [18] and DCFST [20] both employed this method and obtained state-of-the-art performance on multiple benchmarks. Nevertheless, this strategy proposed to generate the reference information only from the first frame, leading to a biased bounding box estimation during the tracking stage and failing to update effectively. Thus, we propose to exploit large-scale tracking datasets to handle the above issues and enhance bounding box estimation by distribution calibration and overlap maximization.

### 2.4. Other Methods

EAST [27] treated the tracking problem as a decision process and selected the optimal policy for bounding box estimation. The scaling action pool is fixed, and thus such estimation is rough. SiamMASK [28] predicted a mask of the target besides the bounding box. However, it has to be trained with extra segmentation datasets and still cannot handle the issues in the direct bounding box regression method. AlphaRefine [32] combined multiple bounding box estimation methods, including those in SiamRPN, SiamCAR, and SiamMASK, to boost the tracking performance, which needs much more training datasets and cannot update online effectively.

## 3. Proposed Approach

In this section, we first provide an overview of the proposed DCOM in Section 3.1. Our bounding box estimation method, DCOM, is composed of three parts, including the overlap maximization module (Section 3.2), the distribution calibration module (Section 3.3), and the updating strategy (Section 3.4). Finally, we discuss the differences between DCOM and other bounding box estimation methods in Section 3.5.

### 3.1. Overview

**Figure 2:**
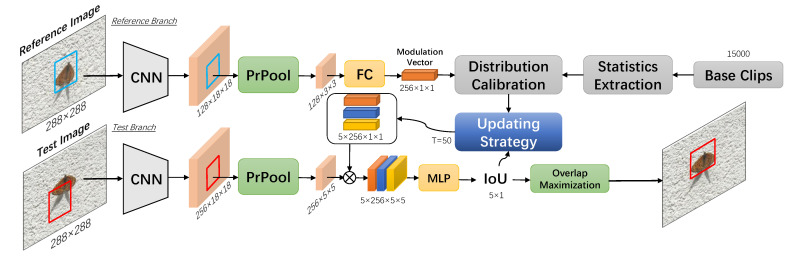
An overview of the proposed DCOM. The CNN module is composed of the backbone network and an extra convolution layer, and the MLP (multi-layer perception) consists of three fully connected layers.

An overview of DCOM is shown in Figure 2. The reference branch receives the reference image and the bounding box of the target object as the inputs, and it outputs a modulation vector as the initial reference information. The distribution calibration module receives the statistics from the base clips and the original modulation vector as inputs, followed by an updating module. The updating strategy generates the final modulation vectors according to the IoU and the calibrated distribution of the reference information. The new modulation vectors are then employed in the test branch to predict the overlap between the candidate box and the groundtruth. The overlap maximization module is used to refine the final bounding box.

### 3.2. Preliminary

Bounding box estimation by overlap maximization [17], which is based on IoU-Net [36], is the baseline of our approach. For the reference branch, given the backbone features $X_{0}$ of the initial frame and the target bounding box annotation $B_{0}$, the method obtains the modulation vector through a convolutional layer, a PrPool layer, and a fully connected layer, that is, $m_{0}=c\left(X_{0},B_{0}\right)$, where $m_{0}\in R^{1\times 1\times D}$. For the test branch, the method first extracts the backbone features Z of the current test frame. Then, given the initial bounding box estimate B generated by the localization branch, the method employs two convolutional layers and a PrPool layer to obtain the feature representation of the target, i.e., F=z(Z,B), where $F\in R^{K\times K\times D}$, and K is the spatial size. F is then modulated by $m_{0}$ through a channel-wise multiplication, generating the target-specific representation for IoU prediction. The baseline finally uses a multi-layer perception (MLP) to obtain the predicted IoU between $B_{0}$ and B. The above process is formulated by

(1) $$\mathrm{IoU}\left(B\right)=\mathrm{MLP}(c\left(X_{0},B_{0}\right)\cdot z\left(Z,B\right)).$$

### 3.3. Bounding Box Estimation by Distribution Calibration

Since the modulation vector in the baseline only depends on the initial frame, the reference information is biased to the initial state of the target and less reliable as the frame grows, especially when the target undergoes severe variations, failing to provide accurate bounding box estimations continuously in online tracking. Therefore, we propose to enhance bounding box estimation with distribution calibration for visual tracking, that is, generating reliable and diverse reference information via distribution calibration.

We take inspiration from few-shot learning with distribution calibration [24] and propose our distribution calibration module over the modulation vector. We assume every dimension in the modulation vectors follows a Gaussian distribution, and from Table 1, we observe that targets of similar classes and close sizes usually share similar mean and variance. Based on such observations, we are able to make use of the statistics from large-scale training datasets with accurate annotations to calibrate the distribution of modulation vectors in online tracking. Based on the new distribution, reliable and sufficient reference information can be obtained directly. Note that modern trackers only use the large-scale tracking datasets for offline training of the networks but cannot take advantage of such groundtruth information in online tracking effectively. On the contrary, for the first time, our approach enables exploiting the large-scale tracking datasets on the online stage for more precise bounding box estimation, which can alleviate the issue of scarcity of data in online tracking.

**Statistics extraction.** Based on the observation from Table 1, targets with similar sizes tend to share similar mean and variance of the feature representations in reference information. Therefore, for each video of the training datasets, we divide the frames into multiple clips according to the target sizes. In each clip, we have

(2) $$|h_{t}-h_{0}|/h_{0}<0.05,\;\left|w_{t}-w_{0}\right|/w_{0}<0.05,$$

where *h* and *w* are the height and width of the target, and $\left[h_{0},w_{0}\right]$ is the target size in the first selected frame of the clip. To avoid noise, clips from all videos, where the frame number is greater than 50, are selected as base clips.

Then, given the annotations, we obtain the modulation vectors of all frames in base clips through the reference branch. The mean of every dimension in the vector for each base clip is calculated as follows:

(3) $$\mu_{i}=\frac{\sum_{j=1}^{n_{i}}m_{j}}{n_{i}},$$

where $n_{i}$ is the frame number of the *i*-th base clip. The covariance matrix $\Sigma_{i}$ for the modulation vectors from the *i*th base clip is given by

(4) $$\Sigma_{i}=\frac{1}{n_{i}-1}\sum_{j=1}^{n_{i}}\left(m_{j}-\mu_{i}\right){\left(m_{j}-\mu_{i}\right)}^{⊤}.$$

**Distribution calibration via statistics transfer.** We obtain the modulation vector of the initial target, $m_{0}$, through the reference branch. Similar to [24], we transform $m_{0}$ into ${\tilde{m}}_{0}$ using Tukey’s ladder of powers transformation [37] to make the distribution more Gaussian-like.Then, we select the top k base clips where the Euclidean distance between ${\tilde{m}}_{0}$ and $\mu_{i}$ is closest. Formally, we have

(5) $$S_{b}=\{i\;|\;topk(\{-\left|\right|\mu_{i}-{\tilde{m}}_{0}{\left|\right|}^{2}\;|\;i\in C_{b}\})\},$$

where $S_{b}$ and $C_{b}$ is the selected set and universe of the base clips, respectively, and topk(·) is the operator to select the top *k* elements from the input set. Finally, we calibrate the mean and covariance of the distribution as follows:

(6) $$\mu^{\prime }=\frac{\sum_{i\in S_{b}}\mu_{i}+{\tilde{m}}_{0}}{k+1},\;\Sigma^{\prime }=\frac{\sum_{i\in S_{b}}\Sigma_{i}}{k}.$$

**Bounding box estimation.** In order to provide sufficient and reliable reference information for precise bounding box estimation, we leverage the calibrated the mean and covariance of the distribution to generate a set of extra modulation vectors by sampling from the calibrated Gaussian distribution as follows:

(7) $$G_{0}={\left\{m_{0,j}\;|\;m_{0,j}∼N\left(\mu^{\prime },\Sigma^{\prime }\right)\right\}}_{j=1}^{M},$$

where *M* is the total number of sampled modulation vectors. For the current test frame, given the coarse target location from the localization branch and target size from the previous frame, we obtain the rough bounding box first and then generate N candidate bounding boxes $B\in R^{N\times 4}$ by adding Gaussian noise to the rough bounding box. Then, the predicted IoUs are obtained by the test branch and the modulation vectors, i.e.,

(8) $$I={\left\{I_{p}\;|\;I_{p}=\mathrm{MLP}\left(m_{p}\cdot z\left(Z,B\right)\right),\;m_{p}\in G_{0}\cup \left\{m_{0}\right\}\right\}}_{p=1}^{M+1},$$

where $I_{p}\in R^{N\times 1}$. For simplicity, we obtain $I={\left[{I_{1}}^{⊤},{I_{2}}^{⊤},...,{I_{M+1}}^{⊤}\right]}^{⊤}$, where $I\in R^{N(M+1)\times 1}$. It is noted that $m_{0}$ always contributes to the prediction since it contains the groundtruth information of the target. The refined bounding boxes $\tilde{B}\in R^{N\times 4}$ is estimated by maximizing each predicted IoU in I w.r.t. B using five gradient ascent iterations with a step length of 1. Finally, based on $\tilde{B}$ and I, we obtain the bounding box estimation by taking the mean of the three bounding boxes with highest IoU, i.e., $\hat{B}\in R^{1\times 4}$ and $\bar{I}\in R$.

### 3.4. Updating Strategy for Reference Information

As the tracking frame grows, the reference information from the initial frame becomes less reliable, especially when the target undergoes severe appearance variations such as deformation, which may cause the drift problem of the tracker. Thus, it is necessary to update the reference information during online tracking. Based on the distribution calibration module, we propose a simple yet effective strategy to update the reference information, i.e., the modulation vectors.

To achieve a good balance between efficiency and accuracy, we update every *T* frames, where *T* is the updating interval. Specifically, given the estimated $\hat{B_{t}}$ and ${\bar{I}}_{t}$ in current test frame *t*, we observe that, though the target can be localized with a high confidence via the localization branch, the predicted bounding box is not precise enough when $\theta_{1}<{\bar{I}}_{t}<\theta_{2}$, where $\theta_{1}$ and $\theta_{2}$ are two thresholds. When ${\bar{I}}_{t}\leq \theta_{1}$, the target can hardly be tracked successfully, and we initialize the reference information with that of initial target, i.e., $m_{0}$. When ${\bar{I}}_{t}\geq \theta_{2}$, the modulation vector is kept unchanged for efficiency. When $\theta_{1}<{\bar{I}}_{t}<\theta_{2}$, based on $\hat{B_{t}}$, we obtain the new modulation vector $m_{t}$ of current frame via the reference branch. Then, we perform distribution calibration w.r.t. $m_{t}$ by substituting $m_{0}$ in Equations (5) and (6). Given the calibrated mean and covariance of new reference information, i.e., $\mu_{t}^{\prime }$ and $\Sigma_{t}^{\prime }$, we update the modulation vectors by sampling from the new Gaussian distribution as follows:

(9) $$G_{t}={\left\{m_{t,j}\;|\;m_{t,j}∼N\left(\mu_{t}^{\prime },\Sigma_{t}^{\prime }\right)\right\}}_{j=1}^{M}.$$

As such, compared with the baseline, we are able to obtain more reliable reference information for robust bounding box estimation in the whole process of visual tracking. Note that, if the modulation vector is updated without the distribution calibration, i.e., $G_{t}$ only contains $m_{t}$, tracking performance will not be improved, since $m_{t}$ based on the estimated $\hat{B_{t}}$ is less reliable. We present the main steps of the updating strategy in Algorithm 1.
**Algorithm 1**: Updating strategy for reference information
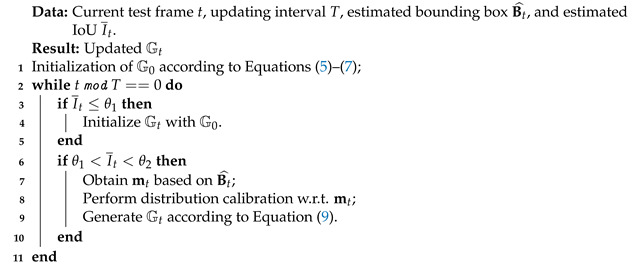



### 3.5. Discussion

**Comparison with direct bounding box regression.** DCOM and the BBR methods are totally different in two aspects. First, BBR methods obtain the estimated box mainly by a regression network/module, which is trained only in the offline process or the first frame, while DCOM obtains the bounding box via an overlap maximization and a distribution calibration module, which benefit from the training datasets in both offline and online process. Second, most BBR methods are tightly coupled with a Siamese-based pipeline, which lacks the process of online discriminative localization, while DCOM is lightweight and can be combined with modern discriminative localization methods easily for robust tracking.

**Comparison with bounding box estimation by overlap maximization.** Although DCOM shares the same overlap maximization module as that of ATOM, they are different in generating and updating reference information. First, ATOM generates the reference information only from the first frame, causing a biased bounding box estimation in online tracking. Second, such reference information is fixed and cannot be updated effectively, since the new reference information provided only by the tracking results is less reliable, and its error will accumlate. To this end, our DCOM improves ATOM in two ways. On the one hand, we make use of the large-scale tracking datasets, which can only be used in offline training in previous methods to provide extra reference information via distribution calibration. On the other hand, DCOM enables a simple yet effective strategy to update reference information according to the updated distribution besides the tracking results. Thus, the reference information in DCOM is more sufficient and less biased for precise bounding box estimation compared with ATOM.

## 4. Experimental Results

To show the universality of our method, we replace the baseline bounding box estimation method in ATOM [17] and DiMP [18] with the proposed DCOM, while keeping the localization branch and the hyperparameter settings unchanged, denoted as ATOM-DCOM and DiMP-DCOM, respectively. We first provide implementation details and then carry out ablation studies to analyse the effect of the sub-modules of our method. Extensive experiments are conducted to evaluate the proposed ATOM-DCOM and DiMP-DCOM and compare their performances against plenty of state-of-the-art trackers on three public benchmarks: LaSOT [22], GOT-10k [21], and NfS [23]. Finally, we provide qualitative comparisons with the baseline trackers.

### 4.1. Implementation Details

ATOM-DCOM and DiMP-DCOM employ ResNet-18 and ResNet-50 [33] as the backbone networks, respectively. Note that our method is built on top of off-the-shelf networks including feature extractor, overlap maximization modules, and localization modules in ATOM [17] and DiMP [18] without extra parameters. We compute in advance and store the statistics for modulation vectors (D=256) from the training sets of GOT-10k [21] and LaSOT [22]. The total number of base clips is 15,000. *M*, *N*, and *k* in Section 3.3 are set to 5, 9, 3, respectively. We set T=50, $\theta_{1}=0.5$, and $\theta_{2}=0.8$ in the updating strategy. Both ATOM-DCOM and DiMP-DCOM is evaluated on three datatsets including LaSOT [22], GOT-10k [21], and NfS [23]. Due to the stochastic nature of DCOM, all results are reported as the average over five runs. We take advantage of the parallel computing in PyTorch to improve the efficiency. On a single Titan RTX GPU, ATOM-DCOM, and DiMP-DCOM achieve real-time speeds of 54 and 38 FPS, respectively.

### 4.2. Ablation Study

We stack the proposed sub-modules, i.e., the distribution calibration module (DC) and the updating strategy (Up) on the baseline, i.e., overlap maximization module, step-to-step to prove the effectiveness of our method. The evaluations are performed on both ATOM-DCOM and DiMP-DCOM on the LaSOT test dataset. Results are shown in Table 3. In Baseline+Noise, we generate $G_{0}$ by adding Gaussian noise to $m_{0}$ instead of DC, causing degraded results. In Baseline+Up, $G_{t}$ only contains $m_{t}\left(t\geq 0\right)$ without extra reference information. This only obtains similar performance to the baseline because bounding box estimation is mainly dependent on $m_{0}$ instead of $m_{t}$, which is not reliable enough. In Baseline+DC, AUC and precision scores are improved by over 1.0% in ATOM-DCOM and over 0.6% in DiMP-DCOM, respectively. The performance is further improved by the updating strategy, confirming the efficacy of the proposed sub-modules.

### 4.3. Results on LaSOT Dataset

**Figure 3:**
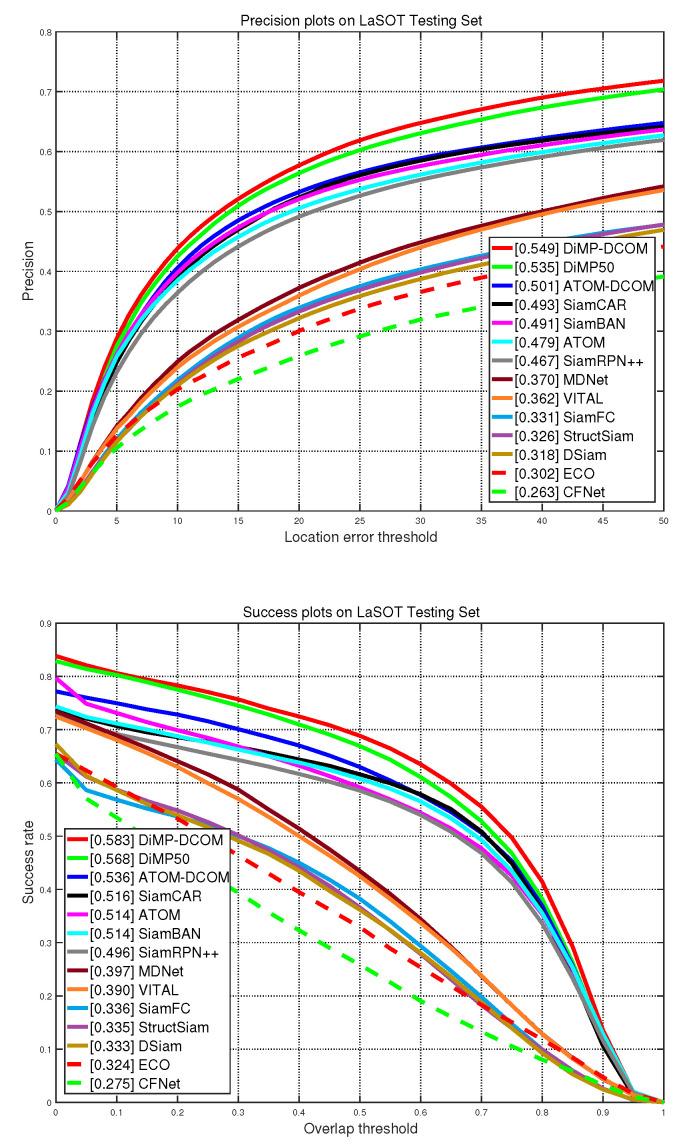
Comparisons with state-of-the-art trackers on LaSOT [22] in terms of precision plots and success plots. All the figures are drawn by the official toolkit.

LaSOT [22] is a large-scale benchmark for long-term single-object tracking. The test set consists of 280 high-quality sequences. The AUC (area-under-the-curve) score and the precision score are listed in Table 4. The success plots and precision plots are shown in Figure 3. ATOM-DCOM and DiMP-DCOM obtain AUC scores of 0.536 and 0.583, respectively, outperforming ATOM and DiMP by 2.1% and 1.5%, respectively. The results show that our bounding box estimation method can bring consistent improvement in terms of AUC and precision scores. Compared with the BBR methods SiamBAN and SiamCAR, DiMP-DCOM shows a large margin of over 6%, confirming that our updating strategy is especially effective in long-term tracking.

### 4.4. Results on GOT-10k Dataset

GOT-10k [21] is a large-scale and high-diversity benchmark for generic object tracking in the wild. Fair comparisons are ensured with the protocol, because all approaches use the same training and testing data provided by the dataset. The evaluation metrics include success plots, average overlap (AO), success rate exceeding 0.5 (${\mathrm{SR}}_{0.5}$), and success rate exceeding 0.75 (${\mathrm{SR}}_{0.75}$). The results are listed in Table 5. ATOM-DCOM and DiMP-DCOM outperform ATOM and DiMP by 1.6% and 1.2% in terms of AO, and 1.7% and 2.0% in terms of ${\mathrm{SR}}_{0.5}$, respectively, showing the effectiveness of our method.

### 4.5. Results on NfS Dataset

**Figure 4:**
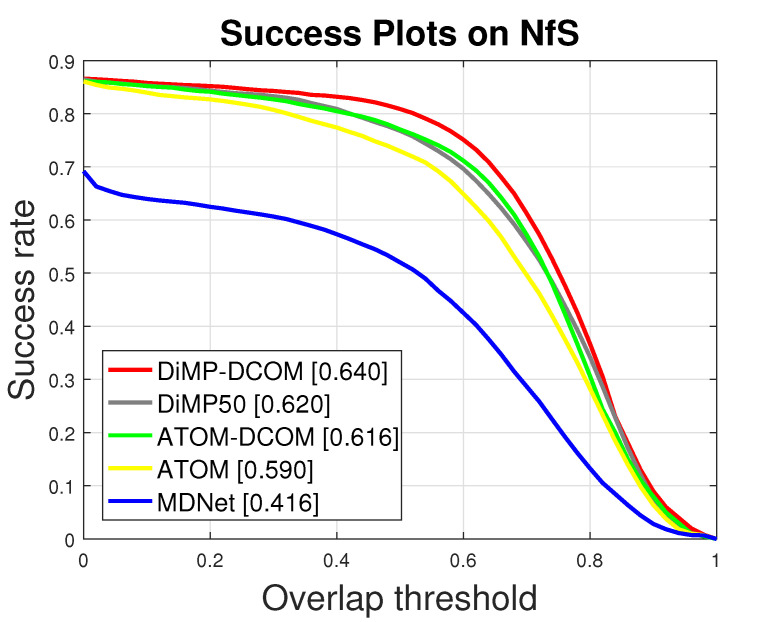
Comparisons with state-of-the-art trackers on NfS [23] in terms of success plots.

We evaluate our approaches on the 30 FPS version of NfS dataset [23], which consists of 100 challenging videos. AUC scores are shown in Table 6 and the success plots are shown in Figure 4. It can be seen that ATOM-DCOM and DiMP-DCOM achieve AUC scores of 0.616 and 0.640, respectively, outperforming ATOM and DiMP by 2.6% and 2.0%, respectively.

### 4.6. Computational Performance

Table 7 lists the mean FPSs of our DiMP-DCOM and ATOM-DCOM and other state-of-the-art trackers on LaSOT. The mFPSs of the other trackers are the same as those in their original papers. The reported tracking speed contains the whole online tracking steps including image loading, feature extraction, target localization, bounding box estimation, and the updating stage. Although both DiMP-DCOM and ATOM-DCOM run 4∼5 FPS slower than their baselines due to the extra computation in distribution calibration, they can still run at real-time speeds. Note that we calculate the statistics of the base clip in advance to avoid double-counting in the online process. We also fully exploit the parallel computing in PyTorch to improve the efficiency when calibrating distribution, generating new reference information and maximizing overlap. DiMP-DCOM runs at a slower speed compared with the BBR methods SiamBAN and SiamCAR, because they lack the process of online update.

### 4.7. Qualitative Results

**Figure 5:**
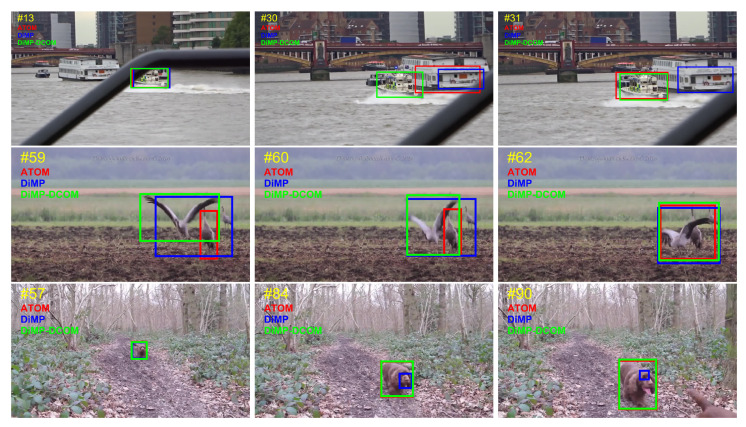

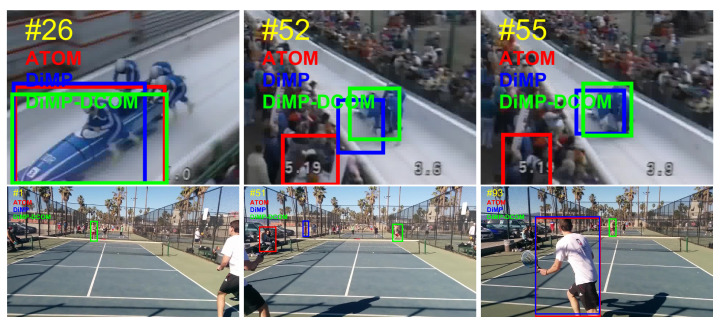
Visualization tracking results of DiMP-DCOM (green), DiMP (blue), and ATOM (red) on the challenging sequences from GOT-10k [21]. We can see that DiMP-DCOM shows stronger ability of bounding box estimation and better accuracy throughout tracking. Best viewed with zooming in.

Although GOT-10k is a short-term benchmark, the variations of the target are severe, and thus it is challenging for precise bounding box estimation; our method provides more sufficient and less biased reference information compared with DiMP and ATOM, leading to a more robust performance. To visualize the bounding box regression quality of our method in online tracking, we show the tracking results of DiMP-DCOM, DiMP [18], and ATOM [17] on the challenging sequences from GOT-10k [21] in Figure 5. Three frames of *GOT-Test-005*, *GOT-Test-018*, *GOT-Test-026*, *GOT-Test-055*, and *GOT-Test-141* sequences are shown in the figures. It can be seen that the bounding boxes of target objects are able to be predicted robustly by DiMP-DCOM when undergoing radical variations, e. g., in *GOT-Test-018*. Note that, in complex scenes such as *GOT-Test-026* and *GOT-Test-141*, DiMP-DCOM will be less impacted negatively by the distractors with the help of the updating strategy, while the other two representative tracking methods, DiMP and ATOM, tend to drift in these scenes only with the limited reference information.

## 5. Conclusions

In this paper, we propose a novel bounding box estimation method for visual tracking, which is based on distribution calibration and overlap maximization. By taking advantage of large-scale training datasets, our method enables generating reliable and diverse reference information during online tracking. Additionally, a simple yet effective updating strategy of the modulation vector is designed for robust online tracking. DCOM is able to improve the state of the art of discriminative trackers by enhancing bounding box estimation. Experiments on three popular benchmarks show the effectiveness of our approach.

## Figures and Tables

**Table 1:** The mean similarity (mSim) and variance similarity (vSim) between the modulation vector of *car-1* the size of 200×125 and those of other target objects from the LaSOT dataset.

Video Name	Target Size	mSim	vSim
*car-6*	200×125	97%	95%
*car-6*	50×40	82%	71%
*car-20*	260×125	92%	88%
*bus-17*	60×50	69%	57%
*boat-8*	200×90	48%	32%
*spider-19*	200×280	36%	14%
*kangaroo-18*	90×205	40%	18%

**Table 2:** Modern trackers and the used bounding box estimation methods.

Tracker	Venue	MSS	BBR	OM	Other
KCF [25]	TPAMI2015				
SAMF [12]	ECCV2014	√			
DSST [13]	TPAMI2017	√			
MDNet [26]	CVPR2016		√		
SiamFC [14]	ECCV2016	√			
ECO [5]	CVPR2017	√			
EAST [27]	ICCV2017				√
SiamRPN [15]	CVPR2018		√		
SiamRPN++ [2]	CVPR2019		√		
SiamMASK [28]	CVPR2019				√
ATOM [17]	CVPR2019			√	
DiMP [18]	ICCV2019			√	
DCFST [20]	ECCV2020			√	
KYS [29]	ECCV2020			√	
SiamCAR [30]	CVPR2020		√		
SiamRCNN [31]	CVPR2020		√		
AlphaRefine [32]	CVPR2021				√

**Table 3:** Ablation study of the sub-modules on LaSOT.

	ATOM-DCOM		DiMP-DCOM	
Method	AUC	Prec.	AUC	Prec.
Baseline	0.515	0.479	0.568	0.535
Baseline+Noise	0.498	0.463	0.542	0.511
Baseline+Up	0.513	0.477	0.568	0.536
Baseline+DC	0.526	0.490	0.574	0.542
Baseline+DC+Up	0.536	0.501	0.583	0.549

**Table 4:** Comparisons with the state-of-the-art trackers on LaSOT.

Tracker	Backbone	AUC	Prec.
ECO [5]	VGG-m	0.324	0.302
MDNet [26]	VGG-m	0.397	0.370
SiamRPN++ [2]	ResNet-50	0.496	0.467
MAML [1]	ResNet-18	0.523	-
SiamCAR [30]	ResNet-50	0.516	0.493
SiamBAN [16]	ResNet-50	0.514	0.491
ATOM [17]	ResNet-18	0.515	0.479
DiMP [18]	ResNet-50	0.568	0.535
ATOM-DCOM	ResNet-18	0.536	0.501
DiMP-DCOM	ResNet-50	**0.583**	**0.549**

**Table 5:** Comparisons with the state-of-the-art trackers on GOT-10k.

	SiamRPN++	SiamCAR	ATOM	DiMP	ATOM-DCOM	DiMP-DCOM
AO	0.517	0.579	0.556	0.611	0.572	**0.623**
SR0.50	0.616	0.677	0.634	0.717	0.651	**0.737**
SR0.75	0.325	0.437	0.402	0.492	0.407	**0.493**

**Table 6:** Comparisons with the state-of-the-art trackers on NfS.

	ECO	SiamRPN++	ATOM	DiMP	ATOM-DCOM	DiMP-DCOM
AUC	0.466	0.620	0.590	0.620	0.616	**0.640**

**Table 7:** The mean FPSs of our DiMP-DCOM and ATOM-DCOM and other state-of-the-art trackers on LaSOT.

Tracker	DiMP-DCOM	DiMP	SiamBAN	SiamCAR	ECO	SiamFC
mFPS	38	43	40	52	6	26
**Tracker**	**ATOM-DCOM**	**ATOM**	**MAML**	**SiamRPN++**	**MDNet**	**CFNet**
mFPS	54	58	42	35	1	36

## Data Availability

Not applicable.
